# Supplementary material for: Naupliar exposure to acute warming does not affect ontogenetic patterns in respiration, body size, or development time in the cosmopolitan copepod Acartia tonsa
Source: PLoS One. 2023 Apr 20;18(4):e0282380. doi: 10.1371/journal.pone.0282380 (PMC10118165; doi:10.1371/journal.pone.0282380)
Supplement: S1 File — (DOCX) [file pone.0282380.s001.docx]

**Supporting Information**

**S1. Example oxygen profile.**

Scaled oxygen profile of a single vial (% initial saturation). Control lines (black) show background change in oxygen determined from two control vials (no copepod included). The copepod line (gold) shows oxygen drawdown from a vial containing a single copepodite. Respiration rates are estimated as the slope of the copepod line after accounting for the average slope of the control lines.

**S2. Kaplan-Meier survival of *Acartia tonsa*.**

Kaplan-Meier survival proportion of actively handled individuals for both experimental groups over time. Different treatments are indicated by different line colors. Shading around each line represents Control CI: ±0.14, S.E. = 0.07. Treatment CI: ±0.18, S.E. = 0.09. CI = confidence interval.

**S3. Cox proportional hazard ratios of *Acartia tonsa* mortality.**

Cox proportional hazard ratios showing effect of treatment (experimental group) and experimental replicate on likelihood of mortality. Sample sizes are indicated below group identities. The hazard ratio (S.E.±0.059) is stated in the third column. P-values are shown alongside each point.

**S1 Table. Stage-specific comparison of body length (mm) between sexes of *Acartia tonsa*.**

Output of post hoc estimated marginal means analysis comparing body length (mm) between sexes at each copepodite stage across the two temperature treatments.

| 95% CI for Mean Difference | | | | | |
| --- | --- | --- | --- | --- | --- |
| Comparison | Estimate | SE | DFn, DFd | t | P |
| C1 F – C1 M | -0.0120 | 0.008 | 1, 168 | -1.587 | **0.0120** |
| C2 F – C2 M | -0.022 | 0.011 | 1, 181 | -1.984 | 0.704 |
| C3 F – C3 M | 0.003 | 0.008 | 1, 171 | 0.359 | 1.000 |
| C4 F – C4 M | 0.017 | 0.009 | 1, 176 | 1.898 | 0.759 |
| C5 F – C5 M | 0.053 | 0.007 | 1, 162 | 7.643 | **<0.001** |
| C6 F – C6 M | 0.097 | 0.007 | 1, 161 | 14.123 | **<0.001** |
|  | | | | | |

**S2 Table. Effects of experimental handling on C6 mass-specific respiration rates.**

Three-way ANOVA comparing C6 mass-specific respiration rates between actively handled and handling control individuals.

| Effect | DFn, DFd | SS | MS | F | P |
| --- | --- | --- | --- | --- | --- |
| Treatment | 1, 55 | 0.002 | 0.002 | 5.065 | 0.156 |
| Handling | 1, 55 | 0.0002 | 0.0002 | 0.498 | 0.295 |
| Sex | 1, 55 | 0.001 | 0.002 | 2.806 | 0.093 |
| Teatment:Handling | 1, 55 | 0.001 | 0.001 | 1.476 | 0.333 |
| Treatment:Sex | 1, 55 | 0.0001 | 0.0001 | 2.546 | 0.074 |
| Handling:Sex | 1, 55 | 0.000 | 0.000 | 0.233 | 0.622 |
| Treatment:Handling:Sex | 1, 55 | 0.0002 | 0.0002 | 0.622 | 0.272 |

**S3 Table. Effects of experimental handling on C6 body lengths (mm).**

Three-way ANOVA comparing C6 body lengths through development between sexes and actively handled versus handling control individuals.

| Effect | DFn, DFd | SS | MS | F | P |
| --- | --- | --- | --- | --- | --- |
| Treatment | 1, 55 | 0.001 | 0.001 | 0.573 | 0.276 |
| Handling | 1, 55 | 0.000 | 0.000 | 0.002 | 0.374 |
| Sex | 1, 55 | 0.103 | 0.103 | 100.3 | **<0.001** |
| Teatment:Handling | 1, 55 | 0.000 | 0.000 | 0.344 | 0.402 |
| Treatment:Sex | 1, 55 | 0.000 | 0.001 | 0.815 | 0.765 |
| Handling:Sex | 1, 55 | 0.000 | 0.000 | 0.452 | 0.984 |
| Treatment:Handling:Sex | 1, 55 | 0.000 | 0.001 | 0.389 | 0.327 |

**S4 Table. Effects of experimental handling on copepodite development time.**

Three-way ANOVA comparing copepodite development time between sexes and actively handled versus handling control individuals.

| Effect | DFn, DFd | SS | MS | F | P |
| --- | --- | --- | --- | --- | --- |
| Treatment | 1, 55 | 2.01 | 2.008 | 1.540 | 0.099 |
| Handling | 1, 55 | 3.03 | 3.030 | 2.325 | 0.092 |
| Sex | 1, 55 | 8.05 | 8.051 | 6.187 | **0.010** |
| Teatment:Handling | 1, 55 | 0.41 | 0.406 | 0.312 | 0.742 |
| Treatment:Sex | 1, 55 | 0.07 | 0.071 | 0.054 | 0.903 |
| Handling:Sex | 1, 55 | 3.89 | 2.890 | 2.218 | 0.283 |
| Treatment:Handling:Sex | 1, 55 | 0.34 | 0.336 | 0.258 | 0.225 |
