# Supplementary material for: Naupliar exposure to acute warming does not affect ontogenetic patterns in respiration, body size, or development time in the cosmopolitan copepod Acartia tonsa
Source: PLoS One. 2023 Apr 20;18(4):e0282380. doi: 10.1371/journal.pone.0282380 (PMC10118165; doi:10.1371/journal.pone.0282380)
Supplement: Attachment — Submitted filename: PONE-D-23-04134_reviewer.pdf [file pone.0282380.s002.pdf]

PLOS ONE

Naupliar exposure to acute warming does not affect ontogenetic patterns in respiration, body size, or development time in the cosmopolitan copepod Acartia tonsa

--Manuscript Draft--

Manuscript Number:	PONE-D-23-04134
Article Type:	Research Article
Full Title:	Naupliar exposure to acute warming does not affect ontogenetic patterns in respiration, body size, or development time in the cosmopolitan copepod Acartia tonsa
Short Title:	Temperature exposure and copepod respiration through development
Corresponding Author:	Matthew Sasaki<br>University of Connecticut<br>Groton, CT UNITED STATES
Keywords:	Copepod; respiration; metabolism; ontogeny; carry-over; plasticity; temperature stress
Abstract:	Short-term, acute warming events are increasing in frequency in across the world's oceans. In short-lived species like most copepods, the duration of these events can bridge within- and between-generational time scales. Yet, it is unclear whether exposure to acute warming during early life stages of copepods causes carryover effects on metabolism through development, which could reduce the amount of energy devoted to growth and affect copepod population dynamics. We exposed nauplii of an ecologically important coastal species, Acartia tonsa, to a 24-hour warming event (control: 18oC; treatment: 28oC), and then tracked individual respiration rate and body length across development, along with the duration of each developmental stage. As expected, we observed a decrease in mass-specific respiration rates as individuals developed. However, exposure to acute warming had no effect on the ontogenetic patterns in per-capita or mass-specific respiration rates, body length, or development time. The lack of these carryover effects through ontogeny suggests within-generational resilience to acute warming in this copepod species.
Order of Authors:	Mathew Holmes-Hackerd
	Matthew Sasaki
	Hans G. Dam
Additional Information:	
**Question**	**Response**
**Financial Disclosure**<br><br>Enter a financial disclosure statement that describes the sources of funding for the work included in this submission. Review the submission guidelines for detailed requirements. View published research articles from *PLOS ONE* for specific examples.<br><br>This statement is required for submission and **will appear in the published article** if the submission is accepted. Please make sure it is accurate.	This research was supported by a National Science Foundation Grant (OCE 1947965) awarded to HGD. The funders had no role in study design, data collection and analysis, decision to publish, or preparation of the manuscript

Powered by Editorial Manager® and ProduXion Manager® from Aries Systems Corporation

**Unfunded studies**<br>Enter: *The author(s) received no specific funding for this work.*<br><br>**Funded studies**<br>Enter a statement with the following details:<br>• Initials of the authors who received each award<br>• Grant numbers awarded to each author<br>• The full name of each funder<br>• URL of each funder website<br>• Did the sponsors or funders play any role in the study design, data collection and analysis, decision to publish, or preparation of the manuscript?<br>• **NO** - Include this sentence at the end of your statement: *The funders had no role in study design, data collection and analysis, decision to publish, or preparation of the manuscript.*<br>• **YES** - Specify the role(s) played.<br><br>* typeset	
**Competing Interests**<br><br>Use the instructions below to enter a competing interest statement for this submission. On behalf of all authors, disclose any competing interests that could be perceived to bias this work—acknowledging all financial support and any other relevant financial or non-financial competing interests.<br><br>This statement is required for submission and **will appear in the published article** if the submission is accepted. Please make sure it is accurate and that any funding sources listed in your Funding Information later in the submission form are also declared in your Financial Disclosure statement.<br><br>View published research articles from *PLOS ONE* for specific examples.	The authors have declared that no competing interests exist.

Powered by Editorial Manager® and ProduXion Manager® from Aries Systems Corporation

**NO authors have competing interests**<br><br>Enter: *The authors have declared that no competing interests exist.*<br><br>**Authors with competing interests**<br><br>Enter competing interest details beginning with this statement:<br><br>*I have read the journal's policy and the authors of this manuscript have the following competing interests: [insert competing interests here]*<br><br>* typeset	
**Ethics Statement**<br><br>Enter an ethics statement for this submission. This statement is required if the study involved:<br><br>• Human participants<br>• Human specimens or tissue<br>• Vertebrate animals or cephalopods<br>• Vertebrate embryos or tissues<br>• Field research<br><br>Write "N/A" if the submission does not require an ethics statement.<br><br>General guidance is provided below. Consult the submission guidelines for detailed instructions. **Make sure that all information entered here is included in the Methods section of the manuscript.**	N/A

**Format for specific study types**<br><br>**Human Subject Research (involving human participants and/or tissue)**<br>• Give the name of the institutional review board or ethics committee that approved the study<br>• Include the approval number and/or a statement indicating approval of this research<br>• Indicate the form of consent obtained (written/oral) or the reason that consent was not obtained (e.g. the data were analyzed anonymously)<br><br>**Animal Research (involving vertebrate animals, embryos or tissues)**<br>• Provide the name of the Institutional Animal Care and Use Committee (IACUC) or other relevant ethics board that reviewed the study protocol, and indicate whether they approved this research or granted a formal waiver of ethical approval<br>• Include an approval number if one was obtained<br>• If the study involved *non-human primates*, add *additional details* about animal welfare and steps taken to ameliorate suffering<br>• If anesthesia, euthanasia, or any kind of animal sacrifice is part of the study, include briefly which substances and/or methods were applied<br><br>**Field Research**<br><br>Include the following details if this study involves the collection of plant, animal, or other materials from a natural setting:<br>• Field permit number<br>• Name of the institution or relevant body that granted permission	
**Data Availability**<br><br>Authors are required to make all data underlying the findings described fully available, without restriction, and from the time of publication. PLOS allows rare exceptions to address legal and ethical concerns. See the PLOS Data Policy and FAQ for detailed information.	Yes - all data are fully available without restriction

A Data Availability Statement describing where the data can be found is required at submission. Your answers to this question constitute the Data Availability Statement and **will be published in the article**, if accepted.<br><br>**Important:** Stating 'data available on request from the author' is not sufficient. If your data are only available upon request, select 'No' for the first question and explain your exceptional situation in the text box.<br><br>Do the authors confirm that all data underlying the findings described in their manuscript are fully available without restriction?	
**Describe where the data may be found in full sentences. If you are copying our sample text, replace any instances of XXX with the appropriate details.**<br><br>• If the data are **held or will be held in a public repository**, include URLs, accession numbers or DOIs. If this information will only be available after acceptance, indicate this by ticking the box below. For example: *All XXX files are available from the XXX database (accession number(s) XXX, XXX.*).<br>• If the data are all contained **within the manuscript and/or Supporting Information files**, enter the following: *All relevant data are within the manuscript and its Supporting Information files.*<br>• If neither of these applies but you are able to provide **details of access elsewhere**, with or without limitations, please do so. For example:<br><br>*Data cannot be shared publicly because of [XXX]. Data are available from the XXX Institutional Data Access / Ethics Committee (contact via XXX) for researchers who meet the criteria for access to confidential data.*<br><br>*The data underlying the results presented in the study are available from (include the name of the third party*	All data and code relevant to this study are available without restriction from a Zenodo repository (https://doi.org/10.5281/zenodo.7434983). In addition to this repository, data and code are actively maintained on GitHub (https://github.com/ZoopEcoEvo/resp_through_dev/tree/v1).

and contact information or URL).<br>• This text is appropriate if the data are owned by a third party and authors do not have permission to share the data.<br><br>* typeset	
Additional data availability information:	

Powered by Editorial Manager® and ProduXion Manager® from Aries Systems Corporation

Manuscript

Click here to access/download;Manuscript;Holmes-Hackerd_manuscript.docx

Title: Naupliar exposure to acute warming does not affect ontogenetic patterns in respiration, body size, or development time in the cosmopolitan copepod *Acartia tonsa*

Authors: Mathew Holmes-Hackerd, Matthew Sasaki, Hans G. Dam

Affiliation: Department of Maine Sciences, University of Connecticut – Avery Point, Groton, Connecticut, United States of America

Abstract

Short-term, acute warming events are increasing in frequency in across the world's oceans. In short-lived species like most copepods, the duration of these events can bridge within- and between-generational time scales. Yet, it is unclear whether exposure to acute warming during early life stages of copepods causes carryover effects on metabolism through development, which could reduce the amount of energy devoted to growth and affect copepod population dynamics. We exposed nauplii of an ecologically important coastal species, *Acartia tonsa*, to a 24-hour warming event (control: 18°C; treatment: 28°C), and then tracked individual respiration rate and body length across development, along with the duration of each developmental stage. As expected, we observed a decrease in mass-specific respiration rates as individuals developed. However, exposure to acute warming had no effect on the ontogenetic patterns in per-capita or mass-specific respiration rates, body length, or development time. The lack of these carryover effects through ontogeny suggests within-generational resilience to acute warming in this copepod species.

Introduction

Copepods are globally distributed and ecologically important in the marine plankton. As primary consumers, copepods represent a crucial link between primary producers and higher trophic levels. As the primary food source for forage fish, copepods have a direct effect on fisheries recruitment [1,2]. Furthermore, copepods are arguably the most abundant pelagic animals [3] and dominate zooplanktonic biomass. Through their production of carbon- and nutrient-rich fecal pellets, and their diel vertical migration, copepods accelerate vertical transport of particulate and dissolved matter into deeper waters [4,5]. Altogether, copepods play a vital role in global carbon and nutrient cycling. A robust understanding of how copepods are affected by increasing temperature is a crucial component of predictions about their various roles under future climate scenarios.

Organismal responses to climate stressors can be broadly separated into two categories: across-generation (evolutionary change) and within-generation (individual variation and phenotypic plasticity). These timescales are intertwined, however, with the latter providing the raw material for evolutionary change [6,7]. Fine-scale observations of within-generation responses to shifts in environmental conditions can, therefore, provide important insights into both the vulnerability and resilience of populations to climate change. Anthropogenic climate change is predicted to cause a rise in ocean temperatures of ~1.5°C over the next 20 years [8], which may have a variety of adverse effects on marine taxa. Perhaps most impacted by this warming will be small ectotherms [9,10]. In addition to rising average temperatures, short-term heat events (e.g. - marine heat waves) are increasing in intensity, duration, and frequency [11, 12]. Increases in temperature over these shorter timescales pose challenges to organisms that are unique from those presented by the long-term increase in average temperature; namely, the

increase in temperature is often more extreme and may occur over within-generation time scales, even in short-lived marine organisms. Given the potentially short timescale of these events, a population's initial response to acute warming is likely to be driven by phenotypic plasticity. Importantly, there may also be variation in individual responses, due to standing genetic diversity within populations. This individual-level variation may shape the impact of increased temperatures on population persistence over short timescales. Outside of low latitudes, most copepods appear to maintain large buffers between environmental and lethal temperatures [13]. As such, understanding how acute sub-lethal exposure to warming affects copepod performance traits is necessary to help predict population dynamics as these events become more frequent. It is also important to separate the effects of acute events from chronic exposure, especially in taxa like copepods where the duration of exposure may extend across developmental stages.

Temperature is known to have strong effects on copepod life history traits [14,15,16]. For example, temperature alone was found to explain >90% of the variance in growth rates of copepods collected from environments ranging from -1.7 to 30.7°C [15]. Similar dependence on temperature is found in other copepod life history traits such as egg production [17] and ingestion rates [18,19]. Copepod metabolism is also highly temperature sensitive [16]. Broadly, metabolism is understood as the sum of all chemical reactions that occur within a living organism because it entails the rate of energy uptake, transformation, and allocation [20]. Metabolism is a key bioenergetics function that provides insight into the control of individual growth [21,22].

Aerobic respiration rates are often used as a proxy for metabolic rates because almost all oxygen consumption is used to support energy production. By focusing on the relationship between temperature and copepod respiration we may therefore obtain an understanding of

temperature effects on copepod metabolism and performance at the whole organism level. Copepod metabolic rates tend to increase with increasing temperature [23, 24]. At high temperatures, additional increases in metabolic rates may stem from the energetic costs associated with mounting the heat shock response [25]. For instance, effects of exposure to elevated temperatures can be buffered by an increase in intracellular concentrations of heat-shock proteins [26]. This may persist, with carryover effects on copepod metabolism, even after the exposure has ended - the upregulation of heat shock protein gene expression in adult *Acartia tonsa*, for example, can persist for up to 30 hours past the warming event [27]. The metabolic implications of this response strategy remain unexplored.

Respiration rate is also often related to body size. Per capita respiration rate, R, scales to body mass, M, as: $R=aM^{3/4}$ [28]. Hence, the weight-specific respiration rate, R/M, decreases with increasing body mass. This pattern holds for copepods [29,30], with some caveats: the pattern accurately describes copepodite, but not naupliar, stages [31]. Despite large relative changes in mass through the six naupliar stages, mass-specific respiration remains relatively constant [31]. This deviates from the expectation of decreasing mass-specific respiration as the nauplii grow. Furthermore, a large increase in metabolism between the final naupliar stages and first copepodite stage has been observed [31], which is again counter to the expectation that mass-specific respiration rate should decrease with increased mass. The stark metabolic differences between copepod life stages suggest there may also be differences in the response to elevated temperature. Generally, early life stages of marine organisms are less tolerant to elevated temperature and thus may act as demographic ‘bottlenecks’ for populations in response to acute warming events (although this is not universal, and copepod nauplii may be more resistant; [32]). Quantifying the effects of exposure to warming during the naupliar stages on

ontogenetic changes in respiration in individual copepods may provide insight into how copepod populations may respond to the increasing threat of short-term sublethal warming events.

The goals of this study are to test the resilience of copepods to acute warming experienced during the naupliar stages, and to quantify potential carryover effects of short-term sublethal warming. We used high sensitivity respirometry to measure individual respiration rates of the calanoid copepod, *Acartia tonsa*, throughout development in response to an initial naupliar warming treatment; thus, providing a fine-scale perspective on the effects of warming exposure on copepod metabolism through development. The temperature treatment used was an increase of +10°C relative to rearing temperature for 24 hours. This timescale was much shorter than the conventional definition of marine heat waves because we were interested specifically in the effects of acute warming; a longer timescale exposure would introduce potential confounding effects of temperature on growth rates (as naupliar stages progress near daily). We hypothesized that naupliar warming treatment would lead to (i) higher energetic requirements and thus higher respiration rates during the subsequent copepodite stages, (ii) delayed development, with warming exposed individuals taking longer to progress through copepodite stages, and (iii) reduced body length across copepodite stages following the naupliar warming treatment. Ultimately, we found that exposure to acute warming in the naupliar stages did not affect any of the three variables measured, suggesting that *Acartia tonsa* nauplii are resilient to short-term warming events, and that effects of extended or repeated exposure may be of greater concern.

Materials and Methods

Experimental organisms

Acartia tonsa nauplii were obtained from a laboratory culture initially collected in July 2021 at Esker Point, Groton, CT, USA (41.3209°N, 71.9996°W). The culture was maintained in an 11-liter container at 18°C in a temperature controlled environmental chamber under a 12:12 light:dark cycle. A small aquarium pump was used to ensure constant aeration of the culture. The culture was regularly fed *ad-libitum* a mixture of *Tetraselmis sp.*, *Rhodomonas sp.*, and *Thalassiosira weissflogii*, as used in previous studies [33]. Phytoplankton cultures were grown in F/2 medium and kept in the exponential growth phase under the same light and temperature conditions as the copepods.

Temperature selection and naupliar respiration rates

Nauplii were exposed to a 28°C warming treatment at the initiation of the experiments. Preliminary experiments showed that this temperature did not exceed naupliar thermal tolerance while still inducing a strong metabolic response, indicating an impactful but sub-lethal heat challenge (Fig 1b). The naupliar survivorship curve was generated following methods previously used for adult *A. tonsa* [33], with individual nauplii survivorship measured after a 24-hour exposure to a range of temperatures. A logistic regression was fit to this individual survivorship data to generate the thermal survivorship curve, which was used to estimate thermal tolerance (LD_{50}, the temperature inducing 50% mortality). Respiration rate was estimated as the rate of oxygen depletion over time (see **Respiration analysis** below for further details). Oxygen measurements were taken using a Presens® oxygen sensor system, which previous studies have used to measure respiration rates of copepod nauplii [34]. In brief, the system uses airtight 2.8 mL vials in a dish atop an optical reader.

Each vial contains a sensor spot made of oxygen-sensitive foil with an immobilized fluorescent dye along the base. These optical sensors measure oxygen concentration within the vial at discrete time intervals. Here we used 3-minute intervals to minimize the slight temperature fluctuations caused by the optical measurements. Naupliar respiration rates were measured for 8 hours in a standing incubator with no light. Three replicate 8-hour trails were conducted at both 18°C and 28°C. Each replicate included 10 vials containing 15 nauplii each and 2 control vials with no nauplii.

Experimental design

Three replicate experiments were performed to test the effect of exposure to acute warming in the naupliar stages through subsequent developmental stages. To initiate each replicate ~200 newly hatched nauplii were isolated from the laboratory culture and evenly split into six 50 mL centrifuge tubes filled with 30 mL of replete food solution (800 µg C/L; 400 µg C/L each of *Tetraselmis* sp. and *Rhodomonas* sp.) These six tubes were randomly assigned to either control (18°C) or heat treatment (28°C) groups (n = 3 tubes per heat treatment per experiment). Each tube was loosely capped and placed into a dry heat bath **(**Thermo Scientific Digital Drybath, Cat# 88870003) and maintained at the assigned temperature treatment for 24- hours.

Following the 24-hour exposure period, 30-40 nauplii were randomly selected from across the tubes (n = 15-20 per treatment) and placed in individual 20 mL petri dishes with replete food solution to monitor development. Petri dishes had been previously cured by soaking in filtered sea water for 7 days with daily seawater replenishment. A subset of these dishes (n = 5-10 each treatment) was randomly selected and assigned to a handling control treatment. Handling controls were monitored daily (see **Daily monitoring and trait measurements**) but trait measurements

were not made until the C6 stage, allowing us to examine potential handling effects on experimental copepods, which were measured and handled more regularly (see **Daily monitoring and trait measurements**). These experiments were blinded, with the treatment assignment process randomized and petri dish labels free of any indication of which treatment (control or warming treatment) the individual was exposed to.

Daily monitoring and trait measurements

Each petri dish was checked using a dissection microscope every 24 hours for copepod survival. Light intensity was kept at the lowest possible level to visualize the copepod while minimizing potential light stress**.** Each surviving individual was carefully transferred into another cured and labeled 20 mL petri dish containing 10 mL fresh replete food solution. Its previous dish and solution were checked for shed molts to determine if or when the individual had progressed to the next development stage and to estimate stage duration (days).

Each individual's respiration was measured once per developmental stage immediately following a molt being found (see Copepodite respiration rates). Following respirometry, the copepodite was photographed using an inverted microscope (again under minimum light exposure) and transferred back to its individual petri dish. Body lengths were estimated from the photographs using ImageJ [35] and then converted into carbon mass (ng C) using an empirical relationship between mass and length [36].

Copepodite respiration rates

Respiration rates of the six copepodite stages (C1-C6) were measured using the same Presens® system used to assess naupliar respiration. For copepodite measurements, single individuals were

placed in each vial with the same *ad libitum* food solution (800 µg C/L evenly split between *Tetraselmis* sp. and *Rhodomonas* sp.) to prevent starvation effects on respiration rates [36,37]. All respiration measurements were made over 8 hours at 18°C in the dark using a standing incubator. Special care was taken to remove all air bubbles from the vials as their presence interferes with the sensor's capability to accurately measure oxygen concentration.

Respiration analysis

Respiration rates were calculated from the time-dependent oxygen concentrations in the vials using the R statistical and computing software environment (version 4.1.3; [38]) and the *respR* package (version 2.0.2; [39]). Respiration rates were calculated against two or three control vials filled with the replete food solution but without a copepod, to account for background changes in oxygen concentration. Respiration rate was then calculated as the slope of oxygen concentration against time, derived from a linear regression. Oxygen depletion within the control vials was negligible in comparison to the depletion within vials containing a copepod, and thus any potential changes in phytoplankton cell abundance due to grazing during the incubation period were not considered (S1 Fig). The first 350 minutes of oxygen measurements were excluded from the regression analyses because of curvature in the oxygen profiles, likely produced while the oxygen sensor spot became saturated or during temperature acclimation between the laboratory space and standing incubator. Excluding this initial time period ensured linearity of the oxygen drawdown, confirmed by visual inspection of the oxygen traces, which is necessary because calculation of respiration rates assumes linear drawdown. The estimated body mass of each copepodite (ng carbon) was used to calculate mass-specific respiration rates (mL oxygen/ hour/ mg carbon).

Hypothesis testing

To test for differences in survival through development between treatment and control groups a Kaplan-Meier survival analysis was conducted. To further explore potential effects of treatment and experimental replicate on survival a Cox proportional hazard model was applied to the data. Separate linear mixed effects models were used to test the effect of the naupliar warming treatment on copepodite traits (size, respiration rate, and total development time) and whether treatment effects depended on sex and life stage. These models included experimental replicate (n = 3) and, within each replicate, the copepod individual as random effects. For copepodite development time (days from C1 to C6), a linear model was used with fixed effects of sex and naupliar warming treatment and random effect of experimental replicate. To test for handling artifacts on the three variables, the respiration rates and body size of handling controls at C6 were compared to the C6 measurements of the serially measured individuals. Copepodite development time was also compared between actively handled and handling control individuals. Post-hoc comparisons of estimated marginal means from mixed effect models were used to test specific differences between groups and explore significant interactions (‘emmeans’ R package, v 1.7.5; [40]). Model assumptions were validated by visually inspecting residual and normal-quantile plots. Several models included a weighted variance structure to account for unequal variances among treatment groups according to Zuur et al, (2009) [41] using the R package ‘nlme’ (version 3.1-160; [42]).

Results

Nauplii had a thermal tolerance value (measured as LD_{50}) of 32ºC (Fig 1a). Naupliar respiration rates increased when exposed to 28°C (treatment) compared to 18°C (control) (treatment: 1.04±0.06, control: 0.29±0.002 mL O_2/hour/individual (mean±S.E.); Fig 1b). This difference in respiration rates yielded a Q_{10} of 3.6, which is greater than typically published estimates of copepod respiration (Q_{10} = 1.8-2.1; [16,43]) consistent with the idea that the warming temperature chosen induced a strong metabolic response but was not lethal to the nauplii.

Fig 1. Nauplii thermal survivorship and mass-specific respiration rates.

(A) Survivorship curve used to estimate naupliar thermal tolerance. Individual survivorship was determined after 24 hours exposed to a range of temperatures. A logistic regression was fit to this data to estimate the survivorship curve and LD_{50}, the temperature inducing 50% mortality. (B) Box plot of mass-specific respiration rates (mL O_2/hour/mg carbon) of nauplii at 18°C and 28°C. Sample size for both temperatures are 30 measurements per temperature. Measurements occurred for 8 hours, nauplii were reared in 18°C and not acclimated to 28°C ahead of time. The box plot reports median value (black line), interquartile range (colored range). Component respiration rate measurements are shown as individual points.

Body length increased through development in both treatment and control copepodites (Fig 2). Individual females grew from 0.33±0.002 mm in C1 to 0.85±0.007 mm in C6, and males grew from 0.35±0.005 mm in C1 to 0.76±0.006 mm in C6 (mean±S.E.). Per capita respiration also increased throughout development (Fig 3a), ranging by a factor of four from C1 to C6. As expected, body length (and thus calculated mass) increased at a higher rate than respiration as copepods developed, resulting in a decrease in mass-specific respiration throughout development (Fig 3b). Survival in both groups was high (S2 Fig), with 80% (control) and 63% (treatment) of individuals reaching maturity, with no significant effect of treatment on survival (Kaplan-Meier test p=0.21, Cox hazard ratio p=0.228; S2 Fig, S3 Fig). Survival was the same in all three experimental replicates (replicate 1 vs. 2 p=0.283, replicate 1 vs. 3 p=0.407, S4 Fig).

Fig 2. Stage specific body lengths of male and female *Acartia tonsa* copepodites.

Body lengths (mm) for individuals from each experimental group at the various copepodite stages. Different treatments are indicated by fill color. Individual body size measurements are shown as points. Box plot components as in Fig 1B.

Fig 3. Per capita and mass-specific respiration rates of *Acartia tonsa.*

(A) Per capita respiration rates (mL O_2/hour/ind.) of pooled nauplii (n) and individual copepodite stages (C1-C6). (B) Mass-specific respiration rates (mL O_2/hour/mg carbon) of pooled nauplii and copepodite stages. Different treatments are indicated by different fill colors. Individual respiration rate measurements are shown as individual points. Boxplot components as describes in Fig 1B.

In contrast to the strong effects of copepodite stage on measured traits, we found no indication of an effect of naupliar warming treatment on mass-specific respiration rates ($p=0.7166$, Table 1), body size ($p=0.1316$, Table 2), or development time ($p=0.1943$, Table 3). Our first hypothesis that naupliar exposure to warming would cause increased mass-specific respiration rates was tested using a linear mixed effect model, which indicated that mass-specific respiration rates varied between development stages ($p<0.0001$), but not between sexes or experimental group (Table 1). Our second hypothesis that, relative to control individuals, body lengths would decrease after naupliar warming exposure was also not supported. The linear mixed effect model for this hypothesis showed that body length varied between stages ($p<0.0001$) and sexes ($p<0.0001$), but not between treatment groups (Table 2). Post hoc tests show males were larger than females in C1 ($p=0.0120$, S1 Table), both sexes were the same size in C2-C4, and females were larger in C5 and C6 (both $p < 0.001$, S1 Table). Finally, the third hypothesis, that total development time would increase in response to naupliar warming treatment, was also not supported (Fig 4, Table 3); however, development time was sex-dependent, with males developing faster ($p<0.0001$, Table 3).

Table 1. ANOVA output of mass-specific linear mixed effects model.

Effect	DFn, DFd	F	P
Stage	5, 145	30.79	**<0.0001**
Sex	1, 35	1.844	0.1048
Treatment	1, 35	4.537	0.7166
Stage:Sex	5, 145	2.261	0.3401
Stage:Treatment	5, 145	0.5515	0.9959
Sex:Treatment	1, 35	0.4737	0.3183
Stage:Sex:Treatment	5, 145	1.014	0.4076

Summary of results from a linear mixed effects model testing the effects of naupliar warming exposure on mass-specific respiration rates. Warming treatment, sex, and copepodite stage and their interactions were included in the model as fixed effects, while experiment replicate and individual copepod were included in the model as random effects.

Table 2. ANOVA output of stage-specific body lengths linear mixed effects model.

Effect	DFn, DFd	F	P
Stage	5, 145	4103	**<0.0001**
Sex	1, 35	30.08	**<0.0001**
Treatment	1, 35	2.424	0.1316
Stage:Sex	5, 145	49.52	**<0.0001**
Stage:Treatment	5, 145	0.4730	0.6094
Sex:Treatment	1, 35	0.7090	0.1476
Stage:Sex:Treatment	5, 145	2.868	0.0976

Summary of results from a linear mixed effects model testing effects of naupliar warming exposure on body lengths. Warming treatment, sex, and copepodite stage and their interactions were included in the model as fixed effects, while experimental replicate and individual copepods were included in the model as random effects.

Table 3. ANOVA output of copepodite development time linear mixed effects model.

Effect	DFn, DFd	F	P
Sex	1, 35	35.38	**<0.0001**
Treatment	1, 35	3.082	0.1943
Sex:Treatment	1, 35	1.195	0.2743

Summary of results from a linear mixed effects model testing effects of naupliar warming exposure on copepodite development time. Warming treatment and sex and their interactions were included in the model as fixed effects, while experimental replicate and individual copepod were included in the model as random effects.

Fig 4. Copepodite development time of male and female *Acartia tonsa*.

Boxplot of total copepodite development time from C1-C6 between experimental groups for both sexes. Box plot components as in Fig 1.

Handling controls were used to explore potential effects of re-occurring experimental handling on respiration and body length. All comparisons between handling groups were for C6 individuals, as neither respiration rates nor body lengths were measured in the intermediate copepodite stages in the handling controls. Three-way ANOVAs indicated no effect of handling on mass-specific respiration rates ($p = 0.295$, S2 Table), body lengths ($p = 0.276$, S3 Table), and copepodite development time ($p = 0.092$, S4 Table).

In summary, copepod respiration increased through development as did body length. Respiration rates increased at a slower rate than mass leading to decreasing mass-specific respiration rates through development. Females progressed through copepodite stages more slowly than males but ultimately reached a larger size. Neither the naupliar warming exposure nor experimental handling had an effect on mass-specific respiration rates, body size, or copepodite development time.

Discussion

Exposure to sublethal warming can have various effects on copepods that linger past the stress event itself. Carryover effects are important to understand because they represent an increased energetic reallocation toward mediation of the stress and away from growth than estimated by just monitoring organismal performance during a stress event. By measuring and tracking individual copepods through their development we were able to explore potential effects of acute naupliar exposure to warming on ontogenetic patterns in metabolism and body size. In general,

these ontogenetic patterns conformed to expectations, with the relative increase in respiration being less than that of body length, leading to an inverse relationship between mass-specific respiration and copepodite stage. Differences between sexes were observed for body size and development time, but not for mass-specific respiration rates. Interestingly, the naupliar warming exposure used here (+10°C for 24 hours) did not affect subsequent respiration rate, body size, or development time, suggesting that *Acartia tonsa* development is resilient to intense but sub-lethal warming exposure.

With no observable difference in respiration through ontogeny between the treatment and the control, our results suggest that there was no effect of acute warming in the naupliar stage on subsequent energy demand. We expected that the observed hardening tactic of upregulating heat shock proteins under increased temperature exposure in *Acartia tonsa* [44] would manifest as a shift in metabolic processes that would be sustained throughout development. We also hypothesized that production of heat shock proteins would divert energy from growth (decreasing body size) or development (increasing stage durations). While we did not measure heat shock protein production, past works show the high capacity for production and upregulation of heat shock proteins in response to temperature exposure in *Acartia tonsa* [27].

The lack of carryover effects of warming exposure on copepodites was surprising given the strong metabolic response of nauplii to the selected high temperature treatment. There are three plausible explanations for these results. First, carryover effects manifested in unmeasured life history traits such as adult survivorship and life span; in this case, carryover effects are unlikely to manifest in sex-specific trait such as reproductive investment (e.g. – egg production) because there was no difference in the effect of high temperature exposure between males and females. A second possible explanation is compensatory feeding. Individuals were provided with

food at replete levels both during and after exposure to high temperatures. However, while compensatory feeding during the copepodite stages may have reduced effects of exposure to the warming treatment on body size and development time, it would likely not have masked potential effects on respiration rate. Any treatment-induced changes in energetic demand may have been compensated for by increased feeding rates in the naupliar stages before molting to C1, however, when respiration measurements were started. *Acartia* species are known to exhibit compensatory feeding in response to reduced nutritional value of prey [45] and salinity shock [46]. Furthermore, the effects of environmental stressors such as combined warming and acidification are weaker at higher food levels [47]. The third explanation is that *Acartia tonsa* nauplii may have high constitutive expression of heat shock proteins to accommodate the large changes associated with rapid growth, molting, and metamorphosis. Constitutively high expression levels of heat shock proteins during the naupliar stage would not only prime individuals to respond to an acute shock, but would also reduce the relative energetic costs associated with mounting the heat shock response in warming exposed nauplii relative to the controls. Strong evidence shows upregulation of heat shock proteins in *A. tonsa* adults and eggs [27,48], but we are not aware of any work that has explored this capacity for nauplii.

The comparison between warming treatment and control nauplii was the primary objective of this project. The second novel contribution of this work, however, is documenting the ontogenetic patterns in respiration, with measurements made on the same individuals at multiple stages through development. This contrasts with the bulk respiration measurements on different individuals at different developmental stages that have been used previously to describe these patterns [31, 36]. By tracking respiration across development, we are able to account for potential differences in developmental patterns between individuals. The usefulness of this

approach is apparent when comparing the mass (mg C) vs. mass-specific respiration (mL O_2/hour/mg C) of individuals across development. The average slope of this relationship was -0.43 ±0.06 (mean ± SE, n=38) but, between individuals, this slope ranged from -1.17 to 0.56. The wide range of observed slopes reinforces how individual-scale respiration measurements are necessary to account for these differences in response to warming exposure. This is especially important when warming events occur on a within-generational time scale as population responses will be shaped by the magnitude of individual variation in temperature sensitivity, and highlights that broad patterns may not be predictive of populations dynamics over shorter timescales [24].

The ¾ power law has long been applied to describe the inverse relationship between respiration and body mass of animals ($R=aM^{3/4}$; [28]). While this is typically examined in the context of across-taxa differences, a similar pattern plays out during development when respiration increases at a slower rate than mass [49]. In this mass-specific case, the expected exponential value is -0.25 [28]. We found an exponential value of -0.45 ($r^2=0.32$, $p<0.0001$), which suggests that weight-specific respiration rate decreased faster with mass in our study than generally observed (Fig 5). Despite the considerable uncertainty in the explained variance in this relationship, a comparison of ontogenetic patterns in respiration will still be useful to identify how the methodology used here compares to previous work. Yet, comparison among studies is often difficult as respiration rate units vary in both scale ($minutes^{-1}$, $hours^{-1}$, $days^{-1}$) and unit (O_2/individual, /unit dry weight, /unit carbon). Additionally, studies in which length is used to estimate weight of individuals often use different conversion factors, making direct comparisons impossible [31,37]. Hence, here we emphasize the comparison of patterns through ontogeny

while understanding that specific respiration rate measurements are highly variable to the specific parameters and organisms tested.

Fig 5. Relationship between mass-specific respiration (mL Oxygen/hour/mg carbon) and copdpodid mass (mg Carbon).

Scatter plot of $\log_{10}$ mass-specific respiration (mL O_2/hour/ng carbon) against mass (mg carbon). Log regression line is described by $y=x^{-0.45}$, $r^2=0.31$, $p < 2.2 \times 10^{-16}$, n=262.

The first component of ontogenetic patterns in respiration to compare is the increase in mass-specific respiration from naupliar to copepodite stages. Previous work has demonstrated a large metabolic increase from the naupliar to copepodite stages [31]. We also observed an increase in mass-specific respiration between naupliar and C1 copepodite stages, which is consistent with the observations of Epp & Lewis (1980) [31] on a tropical freshwater calanoid (*Notodiaptomus venezolanus*). Because of the differences in species and environments, respiration rate comparisons between these studies seem unwarranted. A direct rate comparison of per capita respiration is possible, however, with a study on *Acartia tonsa* respiration rates in response to starvation [37]. This study used a flow-through respirometry technique with 5 adult females per measurement, and found non-starved adult females (C6) respired 0.95±0.17 nL O_2 individual^{-1} min^{-1} (mean±S.E.). This is much smaller than our observed value of 7.75±0.44 nL O_2 individual^{-1} min^{-1} (mean±S.E.) for adult females at 18°C. Although the copepods being compared here are both *Acartia tonsa*, this comparison does not account for differences in body mass (as the rates compared are per capita and not mass-specific respiration). Furthermore, past works show that *Acartia tonsa* from the Baltic region (as used in the previous study; [37]) are generally deeply genetically diverged from *Acartia tonsa* lineages commonly found in the Western Atlantic and thus there may be differences in baseline metabolic rates between population which this comparison cannot account for [50,51]. While direct comparisons remain difficult due to a

variety of factors that cannot be fully accounted for, the patterns we see are overall consistent with previous works.

Direct comparisons of respiration rates with previous studies are difficult because of the differences in unit of measurement, length-weight conversions, and temperature of measurements. We therefore assessed the validity of our mass-specific respiration rates by exploring if respiration rate can meet feeding demands. We focus on adult female rates as they are the most commonly measured group in other studies [27,31,37]. Our experimentally derived mass-specific respiration value of 0.12 mL O_2/mg C/hour equates to 2.88 mL O_2/mg C/day. Oxygen respiration can then be converted into respired carbon using a respiratory quotient (RQ) between 0.8 and 1.0. i.e., respired carbon = respired oxygen * RQ * 12/22.4 [52]. Thus, in our study females respired 1.23 to 1.54 mg C food /mg C copepod /day, which is equivalent to 123%-154% of their body weight in carbon per day. To sustain such costs, the copepods must ingest approximately 3x this amount of carbon per day [53], equivalent to 369% - 462% of their body weight in carbon. Given the average weight of adult females in our study is ~4 µg C (Fig 2), the required amount of carbon ingested per day is 14.76-18.48 µg C, which aligns with prior work that measured ingestion rates of adult female *A. tonsa* at replete food levels between ~10-20 µg C/female/day [54].

This study represents a step toward understanding the full effects of acute naupliar warming exposure on copepod ontogeny. If this experimental design is to be applied to the real-world environment, we recommend additions to the methodology. Primarily, increasing the time of exposure from 1 day to 5 or more days, to match the accepted definition of marine heat waves, which would increase the applicability of this design to real-world warming scenarios. Additionally, incorporating measurements of more life-history traits (e.g., fecundity and egg

hatching success, grazing rate), which would provide a more comprehensive view of the organismal response to acute warming exposure through ontogeny. And finally, replicating this experimental design across food concentrations could highlight compensatory feeding in response to the acute warming exposure as indicated by our conversion of mass-specific respiration rates into ingestion rates.

In summary, our results show that the ecologically important coastal copepod *Acartia tonsa* is metabolically resilient to acute naupliar warming. We observed no carryover effects throughout development of a metabolically impactful naupliar temperature exposure, with no differences in respiration and body size through development or copepodite development time. This resilience may be important for fisheries recruitment and the global biological carbon cycle in a changing climate, as these are powered at least in part by copepod-mediated processes.

Acknowledgements

We thank Catherine Matassa and George McManus for their useful feedback. This research was supported by a National Science Foundation Grant (OCE 1947965) awarded to H. G. Dam.

Data Availability

All data and code relevant to this study are available without restriction from a Zenodo repository (https://doi.org/10.5281/zenodo.7434983). In addition to this repository, data and code are actively maintained on GitHub (https://github.com/ZoopEcoEvo/resp_through_dev/tree/v1).

References

1. Hunter, J. R. Feeding Ecology and predation of marine fish larvae. In Lasker, R., ed.. 1981; 1:34-77. https://swfsc-publications.fisheries.noaa.gov/publications/CR/1981/8122.PDF

2. Castonguay, M., Plourde, S., Robert, D., Runge, J. A., & Fortier, L. Copepod production drives recruitment in a marine fish. Canadian Journal of Fisheries and Aquatic Sciences. 2008 Aug; 65(8): 1528-1531. https://doi.org/10.1139/F08-126

3. Humes, A. G. How many copepods? Ecology and morphology of copepods. 1994; 1-7. https://doi.org/10.1007/978-94-017-1347-4_1.

4. Turner, J. T. Zooplankton fecal pellets, marine snow and sinking phytoplankton blooms. Aquatic Microbial Ecology. 2002; 27(1):57-102 https://doi.org/10.3354/ame027057

5. Longhurst, A. R., & Harrison, W. G. The biological pump: profiles of plankton production and consumption in the upper ocean. Progress in Oceanography.. 1989; 22(1): 47-123. https://doi.org/10.1016/0079-6611(89)90010-4

6. Ghalambor, C. K., McKay, J. K., Carroll, S. P., & Reznick, D. N. Adaptive versus non-adaptive phenotypic plasticity and the potential for contemporary adaptation in new environments. Functional ecology. 2007 Apr 27; 21(3): 394-407. https://doi.org/10.1111/j.1365-2435.2007.01283.x

7. Hoffmann, A. A., & Sgrò, C. M. Climate change and evolutionary adaptation. Nature. 2011 Feb 23; 470(7335): 479-485. https://doi.org/10.1038/nature09670

8. IPCC, 2022: Climate Change 2022: Impacts, Adaptation, and Vulnerability. Contribution of Working Group II to the Sixth Assessment Report of the Intergovernmental Panel on Climate Change [H.-O. Pörtner, D.C. Roberts, M. Tignor, E.S. Poloczanska, K. Mintenbeck, A. Alegría, M. Craig, S. Langsdorf, S. Löschke, V. Möller, A. Okem, B. Rama (eds.)]. Cambridge University Press.

9. Huey RB, Kingsolver JG. Evolution of thermal sensitivity of ectotherm performance. Trends in Ecology and Evolution. 1989;4(5):131–135.

10. Schulte, P. M. The effects of temperature on aerobic metabolism: towards a mechanistic understanding of the responses of ectotherms to a changing environment. The Journal of experimental biology 2015; 218(12): 1856-1866. https://doi.org/10.1242/jeb.118851

11. Oliver, E. C., Donat, M. G., Burrows, M. T., Moore, P. J., Smale, D. A., Alexander, L. V., et al., Longer and more frequent marine heatwaves over the past century. Nature communications 2018 Apr 10; 9(1): 1-12. https://doi.org/10.1038/s41467-018-03732-9

12. Hobday, A. J., Alexander, L. V., Perkins, S. E., Smale, D. A., Straub, S. C., Oliver, E. C. et al. A hierarchical approach to defining marine heatwaves. Progress in Oceanography 2016 Feb 1; 141: 227-238. https://doi.org/10.1016/j.pocean.2015.12.014

13. Sasaki, M. C., & Dam, H. G. Global patterns in copepod thermal tolerance, *Journal of Plankton Research 2021 July 5;* 43(4): 598-609. https://doi.org/10.1093/plankt/fbab044

14. Heinle, D. R. Temperature and zooplankton. Chesapeake Science. 1969 Sep; 10(3): 186-209. https://doi.org/10.2307/1350456

15. Huntley, M. E., & Lopez, M. D. Temperature-dependent production of marine copepods: a global synthesis. The American Naturalist. 1992 Aug 1; 140(2): 201-242. https://doi.org/10.1086/285410

16. Ikeda, T., Kanno, Y., Ozaki, K., & Shinada, A. Metabolic rates of epipelagic marine copepods as a function of body mass and temperature. Marine Biology. 2001 Sep; 139(3): 587-596. https://doi.org/10.1007/s002270100608

17. Sekiguchi, H., McLaren, I. A., & Corkett, C. J. Relationship between growth rate and egg production in the copepod *Acartia clausi hudsonica*. Marine Biology. 1980 Oct [cited 2022 Dec 6]; 58(2): 133-138. https://doi.org/10.1007/BF00396124

18. Dam, H. G., & Peterson, W. T. The effect of temperature on the gut clearance rate constant of planktonic copepods. Journal of Experimental Marine Biology and Ecology. 1988 Nov 10; 123(1): 1-14. https://doi.org/10.1016/0022-0981(88)90105-0

19. Wlodarczyk, E., Durbin, A. G., & Durbin, E. G. Effect of temperature on lower feeding thresholds, gut evacuation rate, and diel feeding behavior in the copepod *Acartia hudsonica*. Marine Ecology Progress Series. 1992 Aug 27; 93-106. https://www.jstor.org/stable/24829923

20. Brown, J. H., Gillooly, J. F., Allen, A. P., Savage, V. M., & West, G. B. Toward a metabolic theory of ecology. Ecology. 2004 Jul 1; 85(7): 1771-1789. https://doi.org/10.1890/03-9000

21. Thor, P. Relationship between specific dynamic action and protein deposition in calanoid copepods. Journal of experimental marine biology and ecology. 2000 Mar 15; 245(2): 171-182. https://doi.org/10.1016/S0022-0981(99)00159-8

22. Thor, P., Cervetto, G., Besiktepe, S., Ribera-Maycas, E., Tang, K. W., & Dam, H. G. Influence of two different green algal diets on specific dynamic action and incorporation of carbon into biochemical fractions in the copepod *Acartia tonsa*. Journal of Plankton Research. 2022 Apr 1; 24(4): 293-300. https://doi.org/10.1093/plankt/24.4.293

23. Ikeda, T., Sano, F., & Yamaguchi, A. Respiration in marine pelagic copepods: a global-bathymetric model. Marine Ecology Progress Series. 2007 Jun 6 . 339: 215-219. https://www.int-res.com/abstracts/meps/v339/p215-219/

24. Heine, K. B., Abebe, A., Wilson, A. E., & Hood, W. R. Copepod respiration increases by 7% per °C increase in temperature: A meta- analysis. Limnology and Oceanography Letters. 2019 Apr 1 . 4(3): 53-61. https://doi.org/10.1002/lol2.10106

25. Hoekstra, L. A., & Montooth, K. L. Inducing extra copies of the Hsp70 gene *in Drosophila melanogaster* increases energetic demand. BMC evolutionary biology. 2014 Mar 19 . 13(1): 1-11. https://doi.org/10.1186/1471-2148-13-68

26. Hochachka, P. W., & Somero, G. N. Biochemical adaptation: mechanism and process in physiological evolution.. Oxford university press; 2002 . 237

27. Rahlff, J., Peters, J., Moyano, M., Pless, O., Claussen, C., Peck, M. A., et al. Short-term molecular and physiological responses to heat stress in neritic copepods *Acartia tonsa* and Eurytemora affinis. Comparative Biochemistry and Physiology Part A: Molecular & Integrative Physiology. 2017 Jan . 203: 348-358. https://doi.org/10.1016/j.cbpa.2016.11.001

28. Glazier, D. S. Beyond the '3/4-power law': variation in the intra-and interspecific scaling of metabolic rate in animals. Biological reviews. 2005 Aug 26; 80(4): 611-662. https://doi.org/10.1017/S1464793105006834

29. Banse, K. Mass-scaled rates of respiration and intrinsic growth in very small invertebrates. Marine Ecology Progress Series. 1982 Sep 15; 281-297. https://www.jstor.org/stable/24814540

30. Kiørboe, T., & Hirst, A. G. Shifts in mass scaling of respiration, feeding, and growth rates across life-form transitions in marine pelagic organisms. The American Naturalist. 2014 Apr; 183(4): E118-E130.

31. Epp, R. W., & Lewis Jr, W. M. The nature and ecological significance of metabolic changes during the life history of copepods. Ecology. 1980 Apr 1; 61(2): 259-264. https://doi.org/10.2307/1935183

32. Tangwancharoen, S., & Burton, R. S. Early life stages are not always the most sensitive: heat stress responses in the copepod Tigriopus californicus. Marine Ecology Progress Series. 2014 Dec 15; 517: 75-83. https://doi.org/10.3354/meps11013

33. Sasaki, M. C., & Dam, H. G. Integrating patterns of thermal tolerance and phenotypic plasticity with population genetics to improve understanding of vulnerability to warming in a widespread copepod. Global change biology. 2019 Aug 26; 25(12): 4147-4164. https://doi.org/10.1111/gcb.14811

34. Köster M., Krause C., Paffenhöfer G. Time-series measurements of oxygen consumption of copepod nauplii. Mar Ecol Prog Ser. 2008 Jan 17 . 353:157-164. https://doi.org/10.3354/meps07185

35. Rasband, W.S., ImageJ, U. S. National Institutes of Health, Bethesda, Maryland, USA, https://imagej.nih.gov/ij/, 1997-2018.

36. Berggreen, U., Hansen, B., & Kiørboe, T. Food size spectra, ingestion and growth of the copepod *Acartia tonsa* during development: Implications for determination of copepod production. Marine biology. 1988 Oct; 99(3): 341-352. https://doi.org/10.1007/BF02112126

37. Thor, P. Elevated respiration rates of the neritic copepod *Acartia tonsa* during recovery from starvation. Journal of experimental marine biology and ecology. 2003 Jan 15; 283(1-2): 133-143. https://doi.org/10.1016/S0022-0981(02)00473-2

38. R Core Team (2022). R: A language and environment for statistical computing. R Foundation for Statistical Computing, Vienna, Austria. URL https://www.R-project.org/.

39. Harianto, J., Carey, N., & Byrne, M. respR—An R package for the manipulation and analysis of respirometry data. Methods in Ecology and Evolution. 2019 Jun 10; 10(6): 912-920. https://doi.org/10.1111/2041-210X.13162

40. Russell V. Lenth (2022). emmeans: Estimated Marginal Means, aka Least-Squares Means. R package version 1.7.5.https://CRAN.R-project.org/package=emmeans

41. Zuur AF, Ieno EN, Walker NJ, Saveliev AA, Smith GM. Mixed effects models and extensions in ecology with R. New York: Springer; 2009 Jan.

42. Pinheiro J, Bates D, R Core Team. *nlme: Linear and Nonlinear Mixed Effects Models*. R package version 3.1-160. 2007 Oct 4; 1-89 p. https://CRAN.R-project.org/package=nlme.

43. Ikeda, T. Relationship between respiration rate and body size in marine plankton animals as a function of the temperature of habitat. Hokkaido University Fisheries Research Bulletin. 1970 Aug; 21(2): 91-112. https://eprints.lib.hokudai.ac.jp/dspace/handle/2115/23417

44. Petkeviciute, E., Kania, P. W., & Skovgaard, A. Genetic responses of the marine copepod *Acartia tonsa* (Dana) to heat shock and epibiont infestation. Aquaculture Reports. 2015 Nov; 2: 10-16. https://doi.org/10.1016/j.aqrep.2015.04.001

45. Siuda, A. N., & Dam, H. G. Effects of omnivory and predator- prey elemental stoichiometry on planktonic trophic interactions. Limnology and Oceanography. 2010 Sep 1; 55(5): 2107-2116. https://doi.org/10.4319/lo.2010.55.5.2107

46. Calliari, D., Borg, M. C. A., Thor, P., Gorokhova, E., & Tiselius, P. Instantaneous salinity reductions affect the survival and feeding rates of the co-occurring copepods *Acartia tonsa* Dana and *A. clausi* Giesbrecht differently. Journal of Experimental Marine Biology and Ecology. 2008 Jul 18; 362(1): 18-25. https://doi.org/10.1016/j.jembe.2008.05.005

47. Brennan, R. S., J.A. deMayo, H.G. Dam, M.B. Finiguerra, H. Baumann, and M.H. Pespeni. Loss of transcriptional plasticity but sustained adaptive capacity after adaptation to global change conditions in a marine copepod. Nat Commun. 2022 Mar 3; 13: 1147. https://doi.org/10.1038/s41467-022-28742-

48. Nilsson, B., Jepsen, P. M., Rewitz, K., & Hansen, B. W. Expression of hsp70 and ferritin in embryos of the copepod *Acartia tonsa* (Dana) during transition between subitaneous and quiescent state. Journal of plankton research. 2014 Mar/Apr; 36(2): 513-522. https://doi.org/10.1093/plankt/fbt099

49. Harrison, F., Jon, Biewener, A., Bernhardt, R., Bernhardt. J.R., Burger, J.R., et al. White Paper: An Integrated Perspective on the Causes of Hypometric Metabolic Scaling in Animals, *Integrative and Comparative Biology. 2022 Nov; 62(5): 1395-1418.* https://doi.org/10.1093/icb/icac136

50. Drillet, G., Goetze, E., Jepsen, P. M., Højgaard, J. K., & Hansen, B. W. Strain-specific vital rates in four *Acartia tonsa* cultures, I: strain origin, genetic differentiation and egg survivorship. Aquaculture. 2008 Jul 2; 280(1-4): 109-116. https://doi.org/10.1016/j.aquaculture.2008.04.010

51. Geburzi, J. C., Heuer, N., Homberger, L., Kabus, J., Moesges, Z., Ovenbeck, et al. An environmental gradient dominates ecological and genetic differentiation of marine invertebrates between the North and Baltic Sea. Ecology and Evolution. 2022 May 20; 12(5): e8868. https://doi.org/10.1002/ece3.8868

52. Omori, M. and T. Ikeda. Methods in Marine Zooplankton Ecology. New York: John Wiley & Sons; 1984. 332 pp.

53. Valiela I. Marine Ecological Processes. New York: Springer-Verlag; 1984. 546 p.

54. Besiktepe, S., & Dam, H. G. Effect of diet on the coupling of ingestion and egg production in the ubiquitous copepod, *Acartia tonsa*. Progress in Oceanography. 2020 Jul; 186: 102346. https://doi.org/10.1016/j.pocean.2020.102346

Figure 1

Click here to access/download;Figure;Fig1.tif

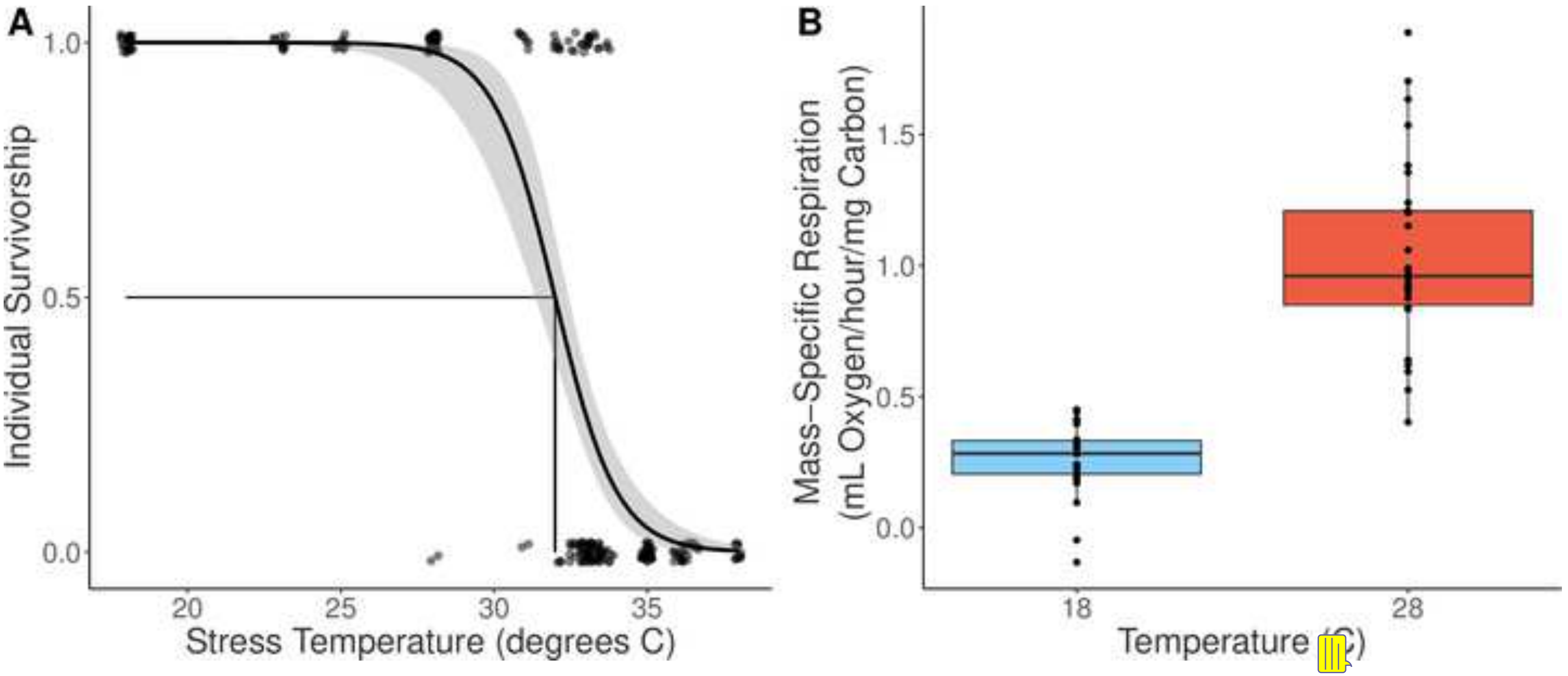

Figure 2

Click here to access/download;Figure;Fig2.tif

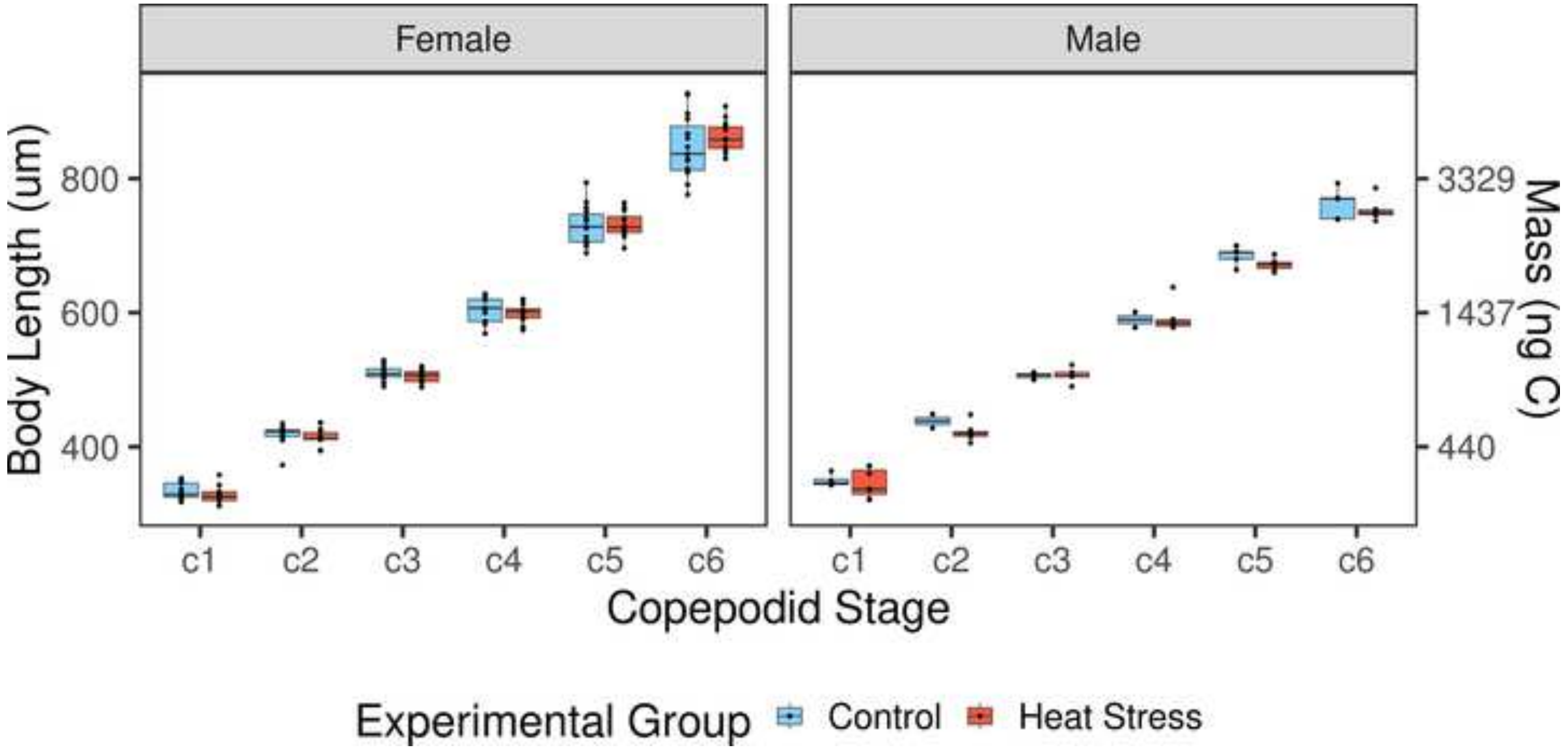

Figure 3

Click here to access/download;Figure;Fig3.tif

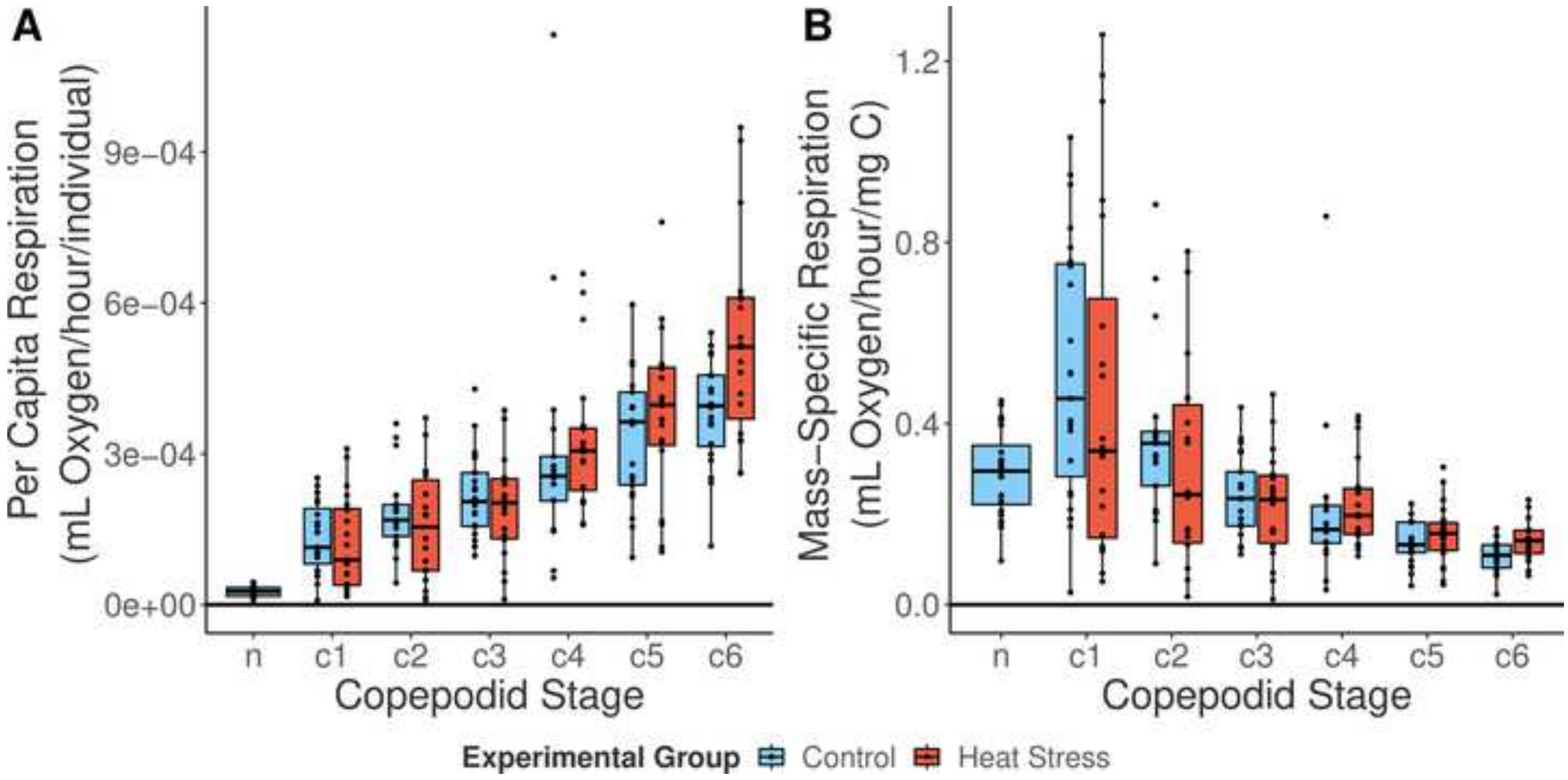

Figure 4

Click here to access/download;Figure;Fig4.tif

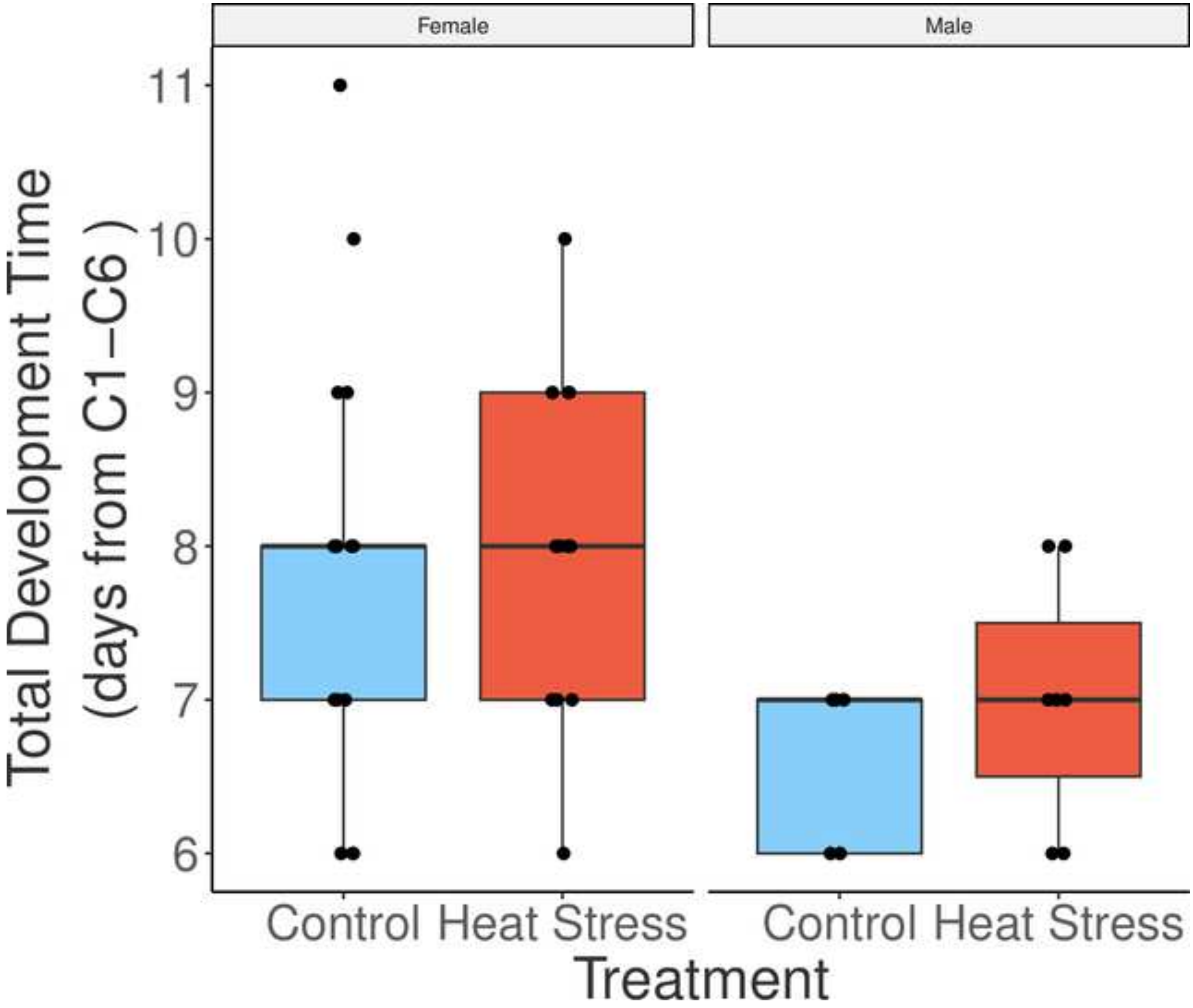

Click here to access/download;Figure;Fig5.tif

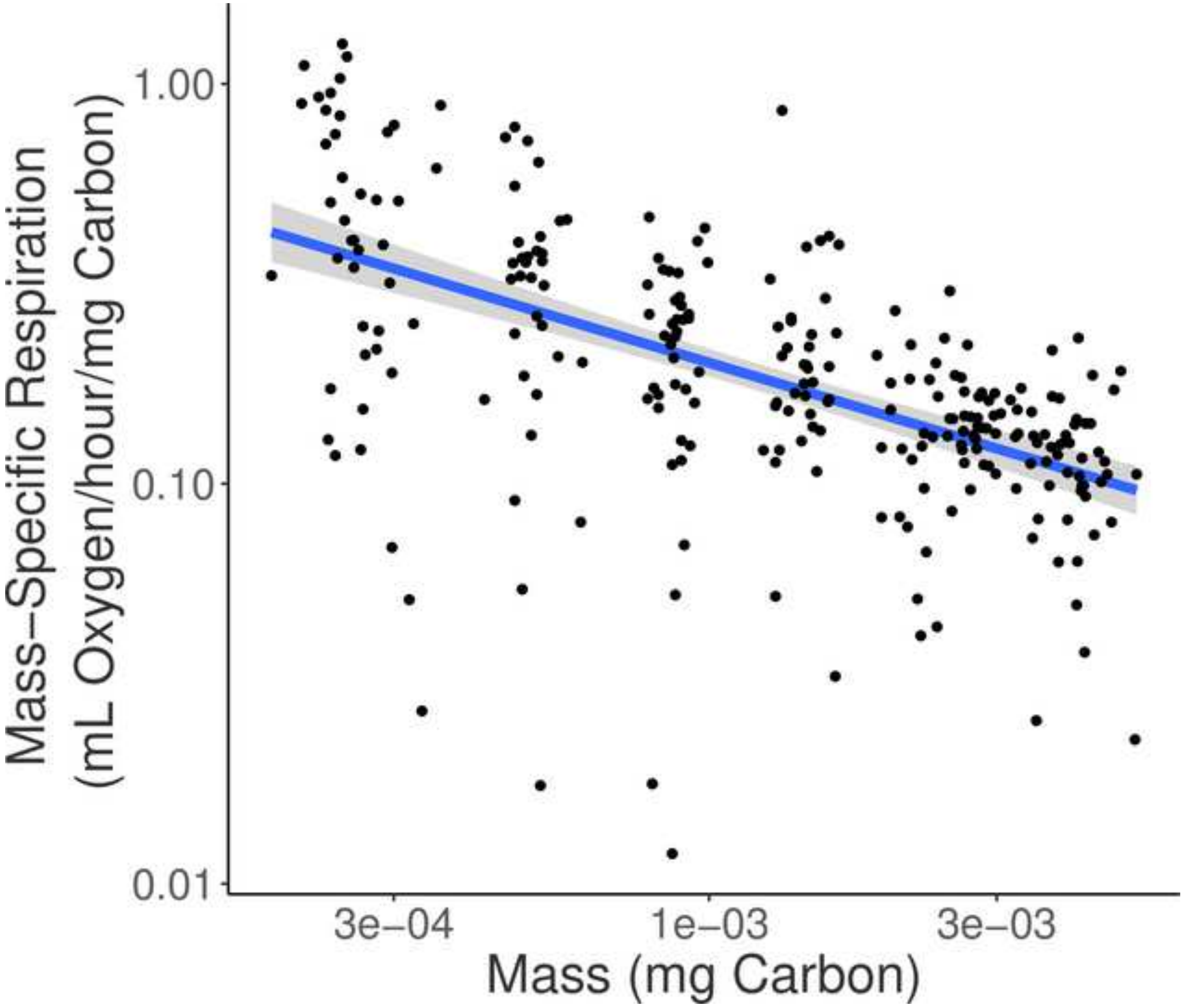

Click here to access/download
Supporting Information
Holmes-Hackerd_SuppInfo.docx

Preprint PDF

Click here to access/download

Preprint PDF

Holmes-Hackerd_preprint.pdf
